# Emojis and Emoticons in Health Care and Dermatology Communication: Narrative Review

**DOI:** 10.2196/33851

**Published:** 2022-08-01

**Authors:** Mindy D Szeto, Cara Barber, Varun K Ranpariya, Jarett Anderson, Jonny Hatch, Jordan Ward, Megan N Aguilera, Shahzeb Hassan, Austin Hamp, Tyler Coolman, Robert P Dellavalle

**Affiliations:** 1 Department of Dermatology University of Colorado Aurora, CO United States; 2 Department of Dermatology Good Samaritan Regional Medical Center/Silver Falls Dermatology Salem, OR United States; 3 Robert Wood Johnson Medical School Rutgers University Piscataway, NJ United States; 4 Abrazo Health Network Goodyear, AZ United States; 5 Texas College of Osteopathic Medicine University of North Texas Health Science Center Fort Worth, TX United States; 6 Paul L Foster School of Medicine Texas Tech University Health Sciences Center El Paso El Paso, TX United States; 7 School of Medicine University of Colorado Anschutz Medical Campus Aurora, CO United States; 8 Feinberg School of Medicine Northwestern University Chicago, IL United States; 9 Department of Dermatology Case Western Reserve University Hospitals Cleveland, OH United States

**Keywords:** emojis, emoticons, dermatology, social media, medicine, public health, COVID-19, narrative review, literature review, mobile phone, Skin of Color

## Abstract

**Background:**

Emoticons and emojis have become staple additions to modern-day communication. These graphical icons are now embedded in daily society through the various forms of popular social media and through users’ personal electronic conversations. With ever-increasing use and inclusivity, exploration of the possible health care and dermatology applications of these tools is imperative.

**Objective:**

The goal of this narrative review was to provide and evaluate an up-to-date literature survey examining the utility of emoticons and emojis in medicine. Special attention was paid to their existing and potential uses in the field of dermatology, especially during the COVID-19 pandemic.

**Methods:**

A PubMed search of peer-reviewed publications was performed in mid-2021 to collect articles with emoticon or emoji keywords in combination with other health care–relevant or dermatology-relevant keywords. Screening of publications and described studies was performed by the authors with education and research experience in health care, dermatology, social media, and electronic communication trends. Selected articles were grouped based on common subjects for qualitative analysis and presentation for in-depth discussion.

**Results:**

From this extensive search, researchers were able to identify a wide variety of publications detailing the use of emoticons and emojis in general health care, pediatric health care, public health, and dermatology. Key subject areas that emerged from the investigation included the ability of emoticons and emojis to improve communication within pediatric health care, enhance mood and psychological assessment or mental health screening in adults, develop interventions to improve patient medication adherence, complement novel means of public health and COVID-19 surveillance, and bolster dermatology-specific applications.

**Conclusions:**

This review illuminated the repurposing of emojis and emoticons for a myriad of advantageous functions in health care and public health, with applications studied in many populations and situations. Dermatology-specific uses were relatively sparse in the literature, highlighting potential opportunities for growth in future studies and practices. The importance of diversity and inclusivity has extended to emojis, with the recent introduction of skin color customization and new emojis better representing the comprehensive spectrum of users’ experiences. A continuously evolving and technology-driven population creates a unique niche for emoticons and emojis to ease worldwide communication and understanding, transcending the barriers of age, language, and background. We encourage future studies and innovations to better understand and expand their utility.

## Introduction

In the ever-evolving world of communication technologies, some of the most popular features include the use of emoticons and emojis, more broadly known as “graphicons” or graphical icons [1]. As electronic communication begins to supplant face-to-face communication, these graphicons can convey emotions and compensate for the lack of nonverbal visual cues in computer-based text, such as facial expressions, body language, and tone of voice. Therefore, emoticons and emojis help broker the relationship between messages and their intended meanings [2].

A portmanteau of “emotion” and “icon,” emoticons specifically refer to icons indicating emotional expressions and were first observed on web-based message boards in 1982. Combinations of keyboard letters and symbols can represent an emotional status by depicting a face or body part, such as “:-D” for laughing, or possibly with other accessories and elements of popular culture, such as “*<\;-)” for Santa Claus [3]. A more recent expansion of the emoticons concept occurred with the development of emojis, defined as “a visual representation of an emotion, idea, or symbolism” and can also enhance text-based and web-based communication [4]. The telecommunications interface designer Shigetaka Kurita devised some of the world’s first emoji sets in the 1990s, drawing inspiration from Japanese pictograms. It was apparent that without a mechanism such as emojis to provide important contextual information, the rise of electronic text communication would be accompanied by an increase in miscommunication. A popular example of an emoji is the “Face with Tears of Joy” (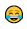), which was the Oxford Dictionary’s Word of the Year in 2015 and remains one of the most commonly used emojis [5]. A recent survey of university students indicated that the overwhelming majority used emojis (91%), most commonly facial expressions, followed by hand gestures, objects, and symbols. They also heavily preferred emojis over emoticons (86%), citing their visual appeal, expressiveness, and ease of use [6]. The use of an image such as an emoji to represent concepts is not a new one. Years of human history have indicated that imagery is an integral portion of language and communication. The ancient Egyptians used pictographic hieroglyphic symbols as their written language to communicate about items, emotions, and stories [7]. Emoji databases presently contain >2823 unique visual representations of different emotions, actions, foods, sports, items, and other concepts, and this number is constantly growing [3]. The concurrent rise of social media has skyrocketed emoji use into a widespread phenomenon, with billions of emojis exchanged daily on different platforms across all genders and nationalities [8].

**Figure 1:**
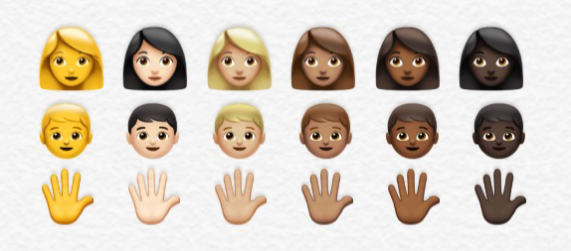
The 6 different skin tone–modifying options for emojis introduced by Apple and the Unicode Consortium in 2015 [11,12].

With the increasing popularity of emoticons and emojis, as well as their established utility in enhancing human communication, the world of health care must consider their influence and role. Effective exchange of information in health care is paramount, and previous studies have indicated that language-based health assessments can often inadvertently perpetuate biases because of language barriers and lower health literacy. Implementation of image-based surveys using emoticons and emojis may be effective in overcoming or even eliminating these potential biases [9]. In addition, physicians should be aware of their use to cater to younger populations and their preferences for using social media, emojis, and texting slang to communicate. Integration of these modalities into regular practice may help forge important communication avenues and rapport between patients and providers [10]. Along these lines, recent movements have sought to increase diversity and inclusivity in the skin tone of emojis to better represent the user; in 2015, the Unicode Consortium, a nonprofit organization upholding international software standards, worked with Apple developers to release an emojis update featuring 6 different skin tone options based on the Fitzpatrick scale in dermatology (Figure 1) [11,12]. However, the potential implications for dermatologic care and Skin of Color dermatology patients remain unclear. Therefore, this narrative review surveys the existing body of scientific literature on the applications of emoticons and emojis in improving various aspects of health care and dermatology, especially in light of the COVID-19 pandemic, triggering further shifts to electronic communication.

## Methods

A PubMed survey of peer-reviewed publications was conducted from May 2021 to December 2021 to identify articles related to emojis and emoticons in the context of health care, dermatology, and the COVID-19 pandemic. PubMed was chosen to conduct the searches as it has been widely recognized as a pre-eminent public source for searching and accessing biomedical literature [13] and currently indexes citations from >34 million publications and 30,000 scientific journals [14]. It was noted that the terms “emoticons” and “emojis” were often conflated and used interchangeably in the literature, despite the subtle differences in definitions we have described in the *Introduction* section [3]. For simplicity, in this paper, we will henceforth use the term “emojis” to refer to the concepts of both “emoticons” and “emojis.” However, to ensure a comprehensive initial screening, we performed literature searches on both terms using combinations of keywords such as “emoticons,” “emojis,” “social media,” “internet,” “dermatology,” “medicine,” “health,” “health care,” “public health,” “covid,” “COVID-19,” and “SARS-CoV-2.” An initial PubMed search of the terms “emojis” and “emoticons” yielded 225 unique publications, which were examined by researchers with education and experience in health care, dermatology, social media research, and trends in electronic communication who independently screened titles and abstracts of search results for relevance and recency, as well as references to important literature cited by resulted publications. Each potential publication required an individual detailed review by the researchers for inclusion, as emoji-specific Medical Subject Headings terms currently do not exist for indexing of PubMed items, and searches returned multiple publications that included only 1 instance of the keyword in the full text, such as research regarding restaurant inspection reports or broad studies of social media sentiment analysis outside of the health care and dermatology scope. Exclusions and subject area determinations were confirmed in consultation with a board-certified dermatologist and a prominent researcher with extensive investigative and editorial experience in health care social media. Preprints, duplicate results, and non–English language publications were also excluded. As our aim was to compile a narrative review of the recent literature, the qualitative analysis focused on examining the specific use of emojis, the populations studied, and the proposed generalizability of the findings. Ultimately, a small subset of articles was featured for in-depth discussion, grouped by a selection of overarching subject areas that emerged from the observed patterns in the results related to applications of emojis.

## Results

### Overview

A selection of 31 recently published articles from studies on general health care, public health, and dermatology was analyzed after screening the emojis literature. We identified several main subject areas, including communication in pediatric health care, assessments of mood and mental health screening in adults, improvements in medication adherence, public health tracking or interventions and COVID-19–related publications, and emoji use in dermatology-specific applications and indicators of skin tone. A narrative review of our findings is detailed in the following sections, organized under the headings of the various article subject groupings. A summary of the study population and type for each examined article is also available in Table 1.

**Table 1:** Summary of relevant emoticon- and emoji-related articles examined in this narrative review grouped by subject area.

Number			Article title		Year published		Study population		Study type		Summary
**Subject Area: Pediatric Health Care**											
	1	Validation of the Wong-Baker FACES Pain Rating Scale in pediatric emergency department patients [15]		2010		120 patients in the emergency department; children aged 8 to 17 years		Prospective observational study		Validation of the Wong-Baker FACES Pain Rating Scale and correlation to a pain severity visual analog scale in children presenting to a suburban academic pediatric emergency department with pain	
	2	Children’s self-report of pain intensity: what we know, where we are headed [16]		2009		50 peer-reviewed publications		Literature review		Synopsis of self-reported measures of pain intensity in children, including an overview of principles, measurement issues, and recommendations for clinical practice and further research	
	3	Use of an animated emoji scale as a novel tool for anxiety assessment in children [17]		2019		102 randomly selected healthy children aged 4 to 14 years visiting an academic pediatric dentistry department in India		Pilot study		Evaluation of a newly designed animated emoji scale to assess dental anxiety in children, with comparisons to the commonly used Venham picture test and facial image scale	
	4	Emoticon use increases plain milk and vegetable purchase in a school cafeteria without adversely affecting total milk purchase [18]		2015		297 children from an inner-city elementary school in Cincinnati, Ohio, United States		Community trial		Investigation of whether emoticon placement next to healthful foods, particularly plain white fat-free milk, in an elementary school cafeteria would increase healthy purchases	
	5	The meaning of emoji to describe food experiences in pre-adolescents [19]		2020		254 preadolescents aged 9 to 13 years attending primary and secondary school in Florence, Italy		Cross-sectional study		Investigation of the emotional meanings and word linkages of emoji used to describe food experiences and analysis of age and gender differences	
	6	Assessing the meaning of emojis for emotional awareness—a pilot study [20]		2019		386 web-based survey respondents		Web-based survey		Investigation of how emotion-related emojis are interpreted by age and gender and assessment of the suitability of emojis in probing for emotional awareness	
	7	Emoji questionnaires can be used with a range of population segments: findings relating to age, gender and frequency of emoji/emoticon use [21]		2018		1084 urban Chinese consumers from diverse demographic and socioeconomic backgrounds		Web-based survey		Assessment of differences in the interpretation of 33 facial emojis and measurement of emotional associations with consumer food products	
	8	Potential of using visual imagery to revolutionise measurement of emotional health [22]		2020		N/A^a^		Commentary		Thought piece exploring how digital visual imagery such as emoji could provide more effective measurements of emotional health	
**Subject Area: Adult Mood and Psychological Assessments**											
	9	Development and preliminary validation of an image-based instrument to assess depressive symptoms [9]		2019		Recruitment via worldwide web-based social media; study 1: 430 young adults; study 2: 482 young adults		Web-based survey		Assessment of depressive symptoms through web-based surveys using 36 emojis; study 1: investigation of participant mood and behavior over the past week, as depicted by emojis, and correlations with the widely used Center for Epidemiologic Studies Depression Scale self-reports; study 2: evaluation of a 10-emoji subset for validity with self-reported depressive symptoms and Big 5 personality traits	
	10	Can an emoji a day keep the doctor away? An explorative mixed-methods feasibility study to develop a self-help app for youth with mental health problems [23]		2019		32 participants aged 16 to 24 years receiving care from a psychiatric facility followed over 3 months		Mixed methods feasibility study		Development and evaluation of a new emoji-based digital mental health daily monitoring tool, G-Moji, to assess positive or negative feelings and allow pattern analyses for potential clinical applications	
	11	Depression screening using daily mental-health ratings from a smartphone application for breast cancer patients [24]		2016		78 adult patients with breast cancer in South Korea, generating 5792 total sets of daily mental health ratings over a 48-week period		Pilot study		Evaluation of an emoji-based mobile mental health daily tracking app to screen for and monitor indicators of depression, with comparisons to PHQ-9^b^ screening	
	12	Sensitivity and specificity analysis: use of emoticon for screening of depression in elderly in Singapore [25]		2018		77 participants aged >65 years recruited from a geriatric outpatient clinic in Singapore		Cross-sectional study		Examination of correlations between mood ratings on an emoji scale and comparisons with DSM-IV^c^ assessments	
	13	Exploring the utility of community-generated social media content for detecting depression: an analytical study on Instagram [26]		2018		749 participants recruited through a web-based crowdsourcing platform		Web-based survey and feature extraction from participants’ Instagram profiles		Investigation of community- and self-generated social media content as a depression screening approach and comparisons with clinically validated PHQ-8^d^ questionnaire responses	
**Subject Area: Medication Adherence**											
	14	Using conversational agents to explain medication instructions to older adults [27]		2018		360 adult participants in the United States recruited from Amazon Mechanical Turk		Web-based survey and pilot study		Development and assessment of a virtual conversational agent system to encourage patient self-care and deliver medication instructions, including an investigation of how appearance, realism, facial cues, and social responses from the virtual agent affect patient learning	
	15	Feasibility and acceptability of a digital health intervention to promote engagement in and adherence to medication for opioid use disorder [28]		2021		24 adult participants undergoing outpatient opioid addiction treatment		Semistructured interviews		Evaluation of the effectiveness, acceptability, and structure of a combined computer-delivered and SMS text message–delivered intervention (including emojis) for individuals initiating buprenorphine treatment for opioid use disorder	
	16	Nudge me: tailoring text messages for prescription adherence through N-of-1 interviews [29]		2021		35 participants with at least one chronic condition treated at a large Colorado health care system		Synchronous video interviews		Evaluation via interviews and content analysis of SMS text messages containing emojis to motivate medication adherence and refills	
**Subject Area: Public Health and the COVID-19 Pandemic**											
	17	Frequencies of private mentions and sharing of mammography and breast cancer terms on Facebook: a pilot study [30]		2017		1.1 million unique female Facebook users generating 1.7 million unique interactions		Cross-sectional study		Analysis of terminology and emoji reactions used in popular social media content regarding breast cancer screening and diagnosis by female Facebook users	
	18	May emoji improve CPR knowledge? [31]		2019		N/A		Commentary		Proposal to add new emojis to the Emoji Unicode List representing the steps of CPR^e^ and early defibrillation to increase awareness and knowledge	
	19	Public awareness, emotional reactions and human mobility in response to the COVID-19 outbreak in China—a population-based ecological study [32]		2020		Mobility data and Weibo social media interactions from 70 million mobile phone users in Sichuan, China		Ecological study		Description and analysis of changes in mobility patterns and public emotional reactions via emojis during the COVID-19 pandemic in early 2020	
	20	Surveilling COVID-19 emotional contagion on Twitter by sentiment analysis [33]		2021		3,308,476 Tweets on Twitter		Focused social media–based sentiment analysis		Examining the flow and content of Tweets, including emojis; exploring the role of COVID-19 pandemic key events, assessing Twitter as a potential surveillance tool for managing pandemic response, and monitoring the spread of information and emotions throughout a population	
	21	COVID-19 and the gendered use of emojis on Twitter: infodemiology study [34]		2020		50,811,299 Tweets from 11,706,754 unique users		Infodemiology study		Analysis of Tweets on Twitter with the hashtags *#Covid19* or *#Covid-19* to determine how emojis were used to discuss various pandemic-related topics and examination of differences in emojis used by gender	
	22	How a smiley protects health: a pilot intervention to improve hand hygiene in hospitals by activating injunctive norms through emoticons [35]		2018		65,907 hand hygiene opportunities and 3340 hand hygiene events at a hospital in Germany		Pilot study		Examination of an emoji-based electronic monitoring and feedback system to reinforce hand sanitizer use by hospital staff in patient rooms, suggesting that activating injunctive norms could improve hand hygiene behavior	
	23	Emojis in public health and how they might be used for hand hygiene and infection prevention and control [36]		2020		57 peer-reviewed publications		Literature review		Overview of emoji use in medicine and public health and how emojis may be used to improve hand hygiene and infection prevention and control	
**Subject Area: Emoji Skin Tone and Dermatology-Specific Applications**											
	24	Technically white: emoji skin-tone modifiers as American technoculture [37]		2019		35 articles, blog posts, videos, podcasts, or opinion pieces published after the introduction of emoji skin tone modifiers and 600 associated user comments		Critical technocultural discourse analysis		Exploration of the significance of emojis and the introduction of emoji skin tone modifiers in terms of race and racial representation and as cultural artifacts where the meaning depends on the cultural and technological context	
	25	The problem with emoji skin tones that no one talks about [38]		2018		N/A		Opinion article		Personal commentary regarding the impact of emoji skin tones on users of various skin tones, suggesting that the 5 possible emoji skin tones still pose limitations and demonstrate a lack of diverse representation	
	26	Linguistic signaling, emojis, and skin tone in trust games [39]		2020		310 undergraduate volunteers		Between-subjects experiment		Assessment of the impact of emoji use, emoji skin tone, and emoji gender on player trust and communication in laboratory-based trust games on mobile devices	
	27	Emoji skin tone modifiers: analyzing variation in usage on social media [40]		2020		80,000 Twitter users		Cross-sectional study		Quantitative and qualitative analysis of variation in the use of emoji skin tone modifiers by different subpopulations of Twitter users and associations with their own real-life skin tone, as well as their choices regarding web-based identity expression and how to represent other users	
	28	The bald emoji effect: alopecia and twitter [41]		2021		1166 tweets, including 808 original tweets		Content analysis		Examination of perceptions of alopecia, hair loss, and related treatments on Twitter; also presenting information about the origin and popularity of the bald emoji	
	29	Social media as a surveillance tool for monitoring of isotretinoin adverse effects [42]		2020		3082 Instagram posts		Cross-sectional study		Analysis of Instagram posts with hashtag *#accutane* to survey public attitudes about oral isotretinoin and adverse effects, which corroborated known side effects and could be used for real-time treatment surveillance	
	30	How do disease perception, treatment features, and dermatologist-patient relationship impact on patients assuming topical treatment? An Italian survey [43]		2015		495 patients with psoriasis at specialized psoriasis hospital centers in Italy		Cross-sectional survey study		Assessment of patient knowledge and attitudes toward psoriasis and treatments via a self-administered questionnaire, including emojis to graphically represent feelings and perceived features of topical therapies	
	31	Validation and banding of the ItchyQuant: a self-report itch severity scale [44]		2017		76 adults with chronic pruritis		Pilot study		Application and investigation of ItchyQuant, an emoji-illustrated numeric rating scale for itch severity, to establish clinical utility, assess patient preferences, and provide validation compared with a traditional numeric rating scale	

^a^N/A: not applicable.

^b^PHQ-9: Patient Health Questionnaire–9 items.

^c^DSM-IV: Diagnostic and Statistical Manual of Mental Disorders, Fourth Edition.

^d^PHQ-8: Patient Health Questionnaire–8 items.

^e^CPR: cardiopulmonary resuscitation.

### Pediatric Health Care

Emojis have been very effective in communicating with children and promoting healthful behaviors. Such visual imagery offers the potential to augment clinical assessment techniques in children or those with cognitive limitations as it can provide information that other forms of communication cannot. Currently, commonly used assessment methods include numeric rating scales, visual analog scales (eg, where participants can indicate subjective pain levels by making a mark along a horizontal line of fixed length), and verbal rating scales. However, in studies of itch and pain severity measurement, these scales were not as suitable and posed difficulties for young children, older adults, and nonnative English-speakers [44]. Thus, emoji-like facial expression illustrations called “faces scales” were developed to address this need. Each facial expression symbolizes a categorical response arranged in an ordinal manner to represent a spectrum of possibilities within a self-reported measure, such as 0 (smiling face, no pain) or 5 (crying sad face, worst pain). Some variations in face scales exist, and research has established that these are generally the pain reporting methods preferred by children [15,16]. Although these face scales could easily be administered on a tablet, at least one of the studies seemed to administer the scales on a paper form [15].

These face scales have been expanded to incorporate motion emoticons and animated emojis to help overcome major barriers in pediatric patient management, such as dental anxiety in children. Fear of dental visits or unwarranted distress over dental procedures is common and may continue into adulthood, contributing to the neglect of oral health. Therefore, early recognition and assessment of dental anxiety is important to identify those needing special assistance or additional support [17]. A comparison of anxiety scales was undertaken to determine an ideal anxiety scale that was easy and efficient to use clinically, appealing, and applicable to younger children with limited cognition and linguistic ability. A newly designed animated emoji scale tested at dental visits for healthy children aged 4 to 14 years showed a high correlation with other common scales, including a face scale; however, the animated emoji scale displayed on an electronic device was the preferred scale by 75% of children and was the expected preference over paper-printed still cartoons [17].

In another successful application of emojis in serving pediatric populations, an inner-city elementary school cafeteria labeled healthy foods with green smiley face emoticons printed on nearby signs and discovered significant increases in children’s selection of plain fat-free milk over chocolate milk, as well as significant elevations in vegetable purchases [18]. Emojis have also been used more generally to help preadolescents describe emotions and experiences associated with food, and gender and age differences were found in how participants discriminated between emojis representing nuances of meaning. Although categories of emotions for children were quite broad initially, they began to narrow during the preschool years, with girls and older children (aged 12-13 years) eventually demonstrating higher levels of understanding when interpreting variations in emotions compared with boys and younger children (aged 9-11 years), particularly when distinguishing different positive expressions [19]. The ability to discriminate among emotions continued to increase with age, whereas gender differences persisted. Familiarity and frequency of emoji use remained higher among women compared with men [19]. However, certain emojis showed greater consensus and high agreement in meaning, such as the aforementioned popular “face with tears of joy” (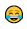), “pouting face” (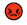), “crying face” (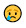), “face with open mouth” or surprised face (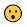), and “neutral face” (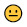) [20]. Furthermore, other studies have found that gender and age differences in the interpretation of emojis became negligible for adults answering emoji questionnaires [21]. Challenges continue to exist surrounding the interpretation of images across different cultures, generations, and demographic groups; thus, further broad investigation is recommended to ensure reliable and valid results in clinical assessments [22].

### Adult Mood and Psychological Assessments

There is also extensive documented use of emojis in mood and psychological assessments for adult populations. Well-validated questionnaires exist for the screening of many conditions such as depression; however, all text-based items that rely on verbal queries are prone to significant bias. Differing education levels and variations in a participant’s primary language can create accessibility barriers to these screening methods [9]. Therefore, nonverbal and image-based approaches that are independent of language, such as emojis, were studied as alternative screening tools. A sample of 482 young adults evaluated an emoji-based 10-item assessment performed on the participants’ PCs or smart devices, with the following directions: “Below is a list of emoji depicting some of the ways you may have felt or behaved. Please indicate if each of the following was true for you much of the time during the past week.” The survey was internally consistent with high sensitivity for screening depression but showed only moderate specificity. Although promising, further validation may be required before truly language-free emoji-based items can replace conventional instruments [9].

The developers of a mobile health app called “G-Moji” extended this approach of using emojis for psychological assessment. In a feasibility study [23], youth and young adults were able to select 1 of the 14 emojis in response to a daily short survey question in the mobile app, “How are you feeling today?” Call logs, location, phone activity levels, app use, social media interactions, and daily routines were passively collected by G-Moji to obtain environmental or sociobehavioral data and contextualize participants’ responses. Participant feedback was used to further improve and develop the apps. All participants agreed that mobile apps such as G-Moji have the potential to build individual awareness of their own behavioral patterns and changes between positive and negative feelings, with the possibility of motivating beneficial lifestyle changes in response. However, these perceived benefits may quickly dissipate for those with severe mental health difficulties, such as struggles with self-harm. Despite this, participants were not observed overall to consider their own feelings more than usual after using the app [23]. Although favorable as a novel way of assessing mental health issues, subsequent investigation of G-Moji is required (with the integration of other collected metrics, which were not analyzed) before its implementation for clinical purposes.

Another mobile mental health daily tracking app was tested in 78 adult patients with breast cancer who reported sleep satisfaction, mood, and anxiety levels as indicators of potential depression over 48 weeks [24]. Participants selected facial emojis arranged on a numeric scale to report their daily ratings of each metric, whereas the validated and commonly used Patient Health Questionnaire (PHQ)–9 items for depression screening was administered biweekly. The performance of the app was comparable with that of the PHQ–9 items screening. Higher adherence to app use was associated with higher screening accuracy. Therefore, accessible and enjoyable approaches to mental health screening demanding minimal cognitive effort, such as emojis, may be less burdensome alternatives for vulnerable participants [24].

Similar findings were observed in a cross-sectional study of mood emoji scales in an older patient population, where hearing impairment or limited language proficiency posed difficulties when other mental health screening methods were used [25]. Participants were asked, “Which of these faces describe your mood over the past 1 week?”; after this question, participants rated their moods using an emoji scale presented by the interviewer. The emoji scale ranged from 1 (most happy face) to 7 (most sad face), which was compared with the Diagnostic and Statistical Manual of Mental Disorders–Fourth Edition (DSM-IV) criteria assessments. Although the sensitivity and specificity of the scale could be improved, it was simple and easy to use, and participants did not exhibit any difficulty in comprehending the questions or differentiating emotions [25].

Emojis can also contribute to the prediction and tracking of depression in communities through social media. A web-based survey study published in the *Journal of Medical Internet Research* recruited 749 participants for depression screening and granted researchers access to their Instagram social media profiles, thereby capturing participants’ posts, captions, and comments [26]. Although previous studies had focused on screening only user-generated social media (including Facebook and Twitter) content created directly by the participant, this study also analyzed community-generated content such as a user’s followers and friends’ responses to the user through received “likes” and comments. Clinically validated PHQ–8 items questionnaire responses were used as reference standards. Various features were extracted from Instagram data to develop a predictive framework with linguistic components, multiple ratings of general user sentiment, and emoji scores. Elastic net regularized linear regression models were then trained to predict PHQ–8 items scores. User-generated and community-generated Instagram content was found to be nonoverlapping, and statistical tests indicated that combining these complementary sources was the most accurate in detecting depression. This suggests that examining social media interactions, including consideration of used emojis, can provide valuable mental health information and play a role in future mental health risk assessment and intervention strategies [26].

### Medication Adherence

It is evident that emojis, whether intentionally created for this purpose, are finding a way into more areas of life than just electronic messaging. Another promising application has emerged in the improvement of health care delivery by increasing medication adherence—the extent to which an individual’s medication use corresponds to their health care provider’s recommendations. As rates of chronic illness and demands of self-care and medication use increase with age, effective modalities for communicating complex medical information and providing instruction to older adults are paramount [27]. On average, medication adherence is quite poor, only approximately 50% for patients with chronic conditions, and is responsible for a substantial proportion of hospital admissions or deaths and health care costs in the United States. Face-to-face communication with health care providers has traditionally been the primary avenue of delivery, especially for older adult patients, which allows the presentation of both verbal and nonverbal (eg, tone of voice and facial expressions) information. This is beneficial for patient retention of instructions [27]. However, inconsistencies in this approach and increasing reliance on digital platforms have led to the exploration of electronically based systems, many of which use emojis.

In one study, a “computer agent” virtual provider was assessed for its ability to deliver medication information to adults [27]. Different levels of realism (photorealism, cartoons, or emojis) in the appearance of the computer agent were tested. Nonverbal and verbal cues were combined in an attempt to elicit social responses from the human participants. Interestingly, the varying degrees of realism were not significantly associated with participants’ memory of the medication messaging, although realistic and cartoon agents received slightly better favorable evaluations than emoji agents and were perceived as more human [27].

The incorporation of emojis into patients’ personalized feedback messages assisted in improving buprenorphine adherence and intervention engagement in a group of 24 adults undergoing outpatient addiction treatment [28]. Trained interviewers surveyed the group for their preferences regarding a new 8-week interactive SMS text message–based digital health program in a qualitative study. Almost all participants reported a desire for the messages to feel more personal by including multimedia elements such as emojis, animations, and videos. Reasons shared included “because then you feel like you're talking to a real person” and “an emoji ruler’s cool ‘cause that’s more eye catchin.’” The more personalized and less generic the messages seemed, the more motivating the intervention was perceived to be. It was also generally acknowledged that younger participants may be more receptive to multimedia features [28].

However, another study found that both younger and older respondents reacted unfavorably when emojis were used in SMS text messages that encouraged timely prescription medication refills [29]. In total, 35 English- and Spanish-speaking patients being treated for at least one chronic condition in a large health care system were interviewed for feedback about the design of the interactive SMS text message intervention. Participants were of diverse ages and ethnic backgrounds and were prompted to choose from different versions of SMS text messages, some of which included emojis, slogans, and variations in the use of abbreviations and message length. Younger respondents noted that the use of emojis felt like the researchers were “trying too hard,” whereas older patients reported feeling confused by emojis [29]. Thus, it appears that further research is needed with larger numbers of participants to elucidate whether using emojis in messages can affect behavior modification strategies surrounding medication adherence.

### Public Health and the COVID-19 Pandemic

Emojis may also offer insights into the perceptions and values of populations when tracking trends in public health. For example, a survey of >1.7 million distinct, breast cancer screening keyword–related Facebook interactions and reactions, including emojis, revealed that 1.1 million unique female Facebook users had contributed in the space of 1 month in 2016 [30]. The most frequently used terms or phrases and shared website links were aggregated according to content, keyword prevalence, age group, and subtotals based on day, among other metrics. The top content category for interactions (36%) was breast cancer–related e-commerce, including both for-profit and nonprofit organization websites selling items connected to breast cancer themes; this content was also the most reshared with users. The second most popular category was celebrity content (26%) commonly originating from television programs, and almost all of these Facebook interactions were emoji reactions to the post. The next largest category was advocacy and charity websites, such as the American Cancer Society donation page. A particular lack of interest in celebrity-driven content was noted among older users, whereas a consistent subgroup of women was responsible for certain popular content with keywords such as “mammogram” [30]. Although this study presented only a limited snapshot of data, it is clear that social media conversations involving emoji reactions and other elements can provide valuable data when attempting to understand current public attitudes and information sources regarding different diseases.

As in previous health care applications, emojis have been used effectively for educational purposes in public health. A proposal to introduce specific new Unicode emojis for cardiopulmonary resuscitation (CPR) was instigated after bystander response time was identified as a crucial factor in improving extrahospital cardiac arrest outcomes [31]. Therefore, given the prevalence of emoji-heavy social media and electronic communication in daily life, the inclusion of new emojis illustrating the CPR rescue chain presents an opportunity to spread awareness about cardiac arrest safety to the public, overcome any language or cultural barriers, and allow for better retention of knowledge. The proposed CPR-related emojis encompassed 6 actions and 2 symbols: an unresponsive person not breathing normally, rescue breaths, 2 emojis depicting chest compression, and 3 indicating the correct sites of defibrillation paddle application along with the presence of an automated external defibrillator and a semiautomatic defibrillator. The addition of these to the Emoji Unicode List would allow the emoji to be used across operating systems and among both electronic and print resources. The easy visualization and cognitive understanding of these symbols have the potential to advance the representation of written and graphical (images and video) information [31].

Emojis have been instrumental in communication during the COVID-19 pandemic, which has affected the lives of nearly everyone, including hundreds of millions worldwide who have been infected by the virus. Emoji tracking of public sentiment during the pandemic was performed, with interesting results. A study of social media in China found that negative emojis were most prominent in January 2020, when the official declaration of human-to-human transmission was made but before the exponential rise in COVID-19 cases occurred. These decreased as COVID-19 cases increased, whereas anger appeared to be expressed most frequently in March 2020 [32]. These emoji data suggest a link between public awareness of the virus and the emotional state of the population, perhaps providing real-time indicators of mental health. Another analysis of the COVID-19 pandemic’s initial emotional impact examined Twitter and categorized the user content as “positive” or “negative.” Similarly, it was noted that Twitter discussions became increasingly negative beginning in January 2020 following the World Health Organization’s report on COVID-19 transmission, and that sentiment was prone to amplification after key events [33]. Demographic differences also exist in pandemic-related emoji social media discourse. An evaluation of 50 million *#Covid-19* and *#Covid19* Twitter posts in 2020 found that although the exchanged emojis generally expressed positive sentiments, the discourse surrounding men was significantly more positive than the discourse surrounding women and sexual or gender minorities [34]. Conversely, emojis referencing death and emergency such as the coffin (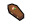), skull (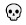), and siren (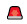) emojis were found much more commonly in male Twitter discourse. The study suggested that this could be related to differences in perceived severity of COVID-19 and higher mortality in men. The laptop (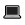) emoji, which was often used to represent changes from in-person to web-based work, was more common in discourse concerning women. Unique gender-specific emojis were also noted, such as the yoga (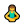), weight lifting (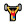), or running (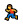) emojis being more frequent in tweets related to women than in those related to men, potentially indicating a higher level of concern by women to exercise and maintain physical health during the pandemic. Therefore, emojis can furnish novel methods for rapid demographic analysis in crisis settings and provide a greater understanding of how emoji use could signify or perpetuate gender roles and differential burdens [34].

Emojis may also assist in hand hygiene, which is a critical element of infection control. Potential tools to improve hand hygiene compliance have explored emojis as part of multimodal educational approaches to simplify instructions, eliminate the need for language translation, and decrease possible misinterpretations of recommendations. Emojis have already been shown to improve hand hygiene behavior in hospital settings. Compared with 3 other tested conditions, an emoticon-based feedback system targeting social norms was found to significantly increase the use of alcohol-based hand rub dispensers [35]. Motion sensors detecting patient room traffic alerted the linked dispenser to possible hand hygiene opportunities for the health care providers. A smiley face (conveying social approval) was displayed on the dispenser’s electronic screen when the provider used the dispenser, whereas a frowny face (social disapproval) appeared when the provider did not. Instant feedback and constant monitoring with visual cues were effective in modifying the behaviors. Moreover, dispensers modified to include this electronic emoji screen were used more than twice as much as dispensers in other rooms [35]. However, adherence to simple hygiene procedures was generally difficult, despite numerous intervention strategies attempting to overcome behavioral obstacles [36]. Currently, there is no emoji that directly shows hand washing or hand sanitization. Using a sequence of existing emojis such as “clapping hands” (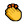) and “bar of soap” (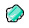) can be cumbersome and create confusing misinterpretations such as applauding the use of soap. Introducing new highly specific emojis may be helpful in the universal dissemination of infection prevention education [36].

### Emoji Skin Tone and Dermatology-Specific Applications

Despite recent efforts to increase diversity and inclusivity through emojis that represent different skin tones, dermatology-specific applications of emojis have been relatively rare. Early emoji sets faced intense backlash from users with Skin of Color because of the marked absence of diverse emoji characters depicting human figures or body parts. Emojis showed largely only White skin tones and, later, “non-human” unnatural yellow skin colors with no customization options. The resulting public pressure eventually prompted Apple and the Unicode Consortium to release an update in 2015 adding skin tone modifiers (Figure 1). The “blank” emoji without skin tone modification is a yellow nonclassified tone, whereas the lightest option is meant to encompass the Fitzpatrick skin type 1 or 2. The remaining options denote one of the Fitzpatrick skin types from 3 to 6. Hair texture options were subsequently expanded in 2018 [37]. However, it has been suggested that adding an element of race or ethnicity to emojis posed a disruption of any original intention to be “raceless” neutral symbols and a requirement of shared cultural context for interpretability [37]. It was also speculated that the new explicit visibility of “whiteness” in emojis created tension for users, regardless of racial self-identity. Some users argued that allowing people to “opt in” for skin tones was never a good solution for true representation. Layering a skin tone on top of a previously designed emoji was construed as akin to white emojis simply “wearing masks” [38]. Concerningly, recent experiments have found that although emojis increased trust among players of a mobile messaging trust game, both light skin and dark skin recipients of dark skin emojis reported significant decreases in trust, suggesting that complex social judgments can be associated with emoji use [39]. However, another study found that using opposite-toned emojis on Twitter demonstrated no evidence of negative racial sentiment. The overwhelming majority of used emoji skin tones matched the skin tone of the user’s profile photo, and users with darker-skinned profile photos were more likely to use emoji skin tone modifiers overall [40]. Thus, although skin tone emojis have attempted to bolster representation, they have also created an avenue for asking difficult questions about what it means to perform a certain identity with emojis and address the intrinsic power dynamics triggered by their use [37].

Nevertheless, the ability to self-identify skin color holds great potential for patient care in dermatology. It has been established that emojis validating a user’s life experiences can be powerful tools for conveying shared emotions and vulnerability. In 2018, a patient with alopecia areata aged 24 years initiated a petition for new emojis to capture her thoughts and feelings, stating “emoji are often used when you don’t know the words to say and when you suffer from hair loss it’s hard to express yourself...if people were able to use one, it would speak volumes” [41]. The bald emoji (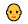) was introduced and became the most popular new emoji of 2018, suggesting that this sentiment was strongly shared by other patients with alopecia who now felt more included on social media platforms [41]. This led to a surge of tweets on the topic, most of which were related to personal experiences, and also educated users about the condition and its symptoms. However, alopecia advertisements promoting hair growth products, wigs, and hair transplantation were also common, potentially propagating misleading treatment information [41].

Although users should approach accessing social media for medical information with caution, user-generated content, including emojis, may be used to monitor the side effects of dermatologic treatment. Although they did not specifically examine emojis, one study analyzed Instagram posts related to the hashtag *#accutane* and identified users of the medication. The social media–reported side effects of the drug were similar to the known side effects, as well as the general pattern of the treatment’s adverse events. Therefore, emojis in dermatology social media posts can be used as expressive tools for real-time treatment surveillance [42]. Another area in which emojis can be used in dermatology is for patients to describe their feelings regarding treatment in a clinical setting. Self-administered questionnaires from hundreds of patients with psoriasis at specialized hospitals in Italy revealed that emoticons helped patients express the therapeutic features that were perceived as the most important or distressing. Various emoticons corresponding to descriptors, such as “soothing,” “reliable,” “greased,” “bedaubing,” and “sticky,” were used with respect to different topical therapy formulations [43]. In similar studies on chronic pruritis, patients of dermatology preferred self-reporting their itch symptoms with a cartoon emoji-based scale called “ItchyQuant” over a purely numerical scale or other quantitative scales. ItchyQuant was administered at either the beginning or end of the patient’s clinic visit. The cartoons represented an increasing amount of itch by the changing facial expressions and the amount of scratching. The ItchyQuant measures demonstrated high concurrence with traditional itch severity scales and were clinically meaningful [44]. Given the substantial negative emotional and psychosocial effects of chronic pruritus, emojis could be valuable tools for assessing challenging dermatologic populations with communication barriers, as was the case in other health care fields. Dermatologists should be aware of emoji applications when approaching different conditions that pertain to their patients to navigate the best personalized care for their dermatological needs.

## Discussion

### Principal Findings

Clearly, emojis can be leveraged and repurposed to fulfill different needs in health care settings. Using emojis can help foster better interactions, including patient-provider relationships, and aid in meeting patients at their preferred level of understanding or cognition. We must take advantage of these novel means to better communicate with patients, as communication is often the largest obstacle to appropriate and comprehensive care, aside from cost [45]. Although imagery such as emojis has been found to yield reliable results in clinical assessments, challenges still exist surrounding their interpretation across cultures and age groups. Therefore, a broad investigative task is ahead to gain a firmer understanding of this and any evolving elasticity of their meaning to different populations [22]. The limitations of our narrative review include the exploratory nature of the search and general exclusion of publications and journals not indexed in PubMed. For example, an important study in a psychological journal not indexed by PubMed [46] by the authors of previously discussed work [9] established the rationale for the suggested linkage of an individual’s self-identification with various emojis and correlations with certain Big 5 personality traits. This highlights another potential use of emojis in the health care space, and future reviews should build upon our initial survey to assess a broader scope of literature.

Nevertheless, current applications of emojis in dermatology are relatively sparse, which is disappointing, given their extensive potential. Our study found that the main clinical applications for emojis specifically within the field of dermatology were limited to expressing attitudes regarding topicals for psoriasis [43] and using emoji-based cartoons to self-report pruritus severity using the ItchyQuant scale [44]. With recent skin tone modifier updates, patients of dermatology could use emojis to self-identify their skin color and help translate their selection into an accurate Fitzpatrick skin type classification, as this was the basis for Apple’s skin tone options. As was performed with ItchyQuant, emojis could be incorporated into practice during pediatric dermatologic encounters for itch and pain, among other symptoms. This could create a more positive and friendly environment for patients, as it can be challenging to communicate with pediatric patients effectively. The psychosocial and emotional aspects of a pediatric or adult patient’s experience could be easily screened at the dermatology office, perhaps using one of the aforementioned mental health–oriented applications [9]. Patients would be able to self-select emojis that describe their mood or thoughts about their condition and treatment. Going a step further, emojis could also be used as a means of patient satisfaction ranking. As health care expectations, reimbursement, and improvements increasingly focus on the quality of care received, offering an accessible variety of emojis to collect feedback quickly could be effective. Difficult or complex dermatology medication instructions and routes of administration can also be simplified using emojis, for example, assigning day or night use, as well as body area and frequency.

As the world continues to progress through a pandemic, forcing many people to turn to virtual formats and telemedicine as safer choices, more advancements in these communication methods must be implemented and used. There is potential for providers and patients to communicate through emoji-enhanced messages, and in dermatology, emojis can complement messaging about lesions, colors, and symptoms, allowing ancillary information to be sent along with chief concerns. However, although the flexibility and ease of emojis may account for a large part of their appeal [5], emoji use in health care communication may also trigger potential medicolegal implications because of their inherent ambiguity of meaning.

With the continued growth of electronic communication, new applications for emojis may emerge. Although our survey was limited by the simplified search strategy highlighting only a few overarching subjects in previous literature, the dynamic rapid nature of social media and internet trends also entails that this growth will inevitably outpace scientific publications. This solidifies the need for subsequent periodic surveys of emoji-focused studies such as this one. The landscape of emojis is also dynamic, and new emojis, such as skin tone customizations and bald emojis, are continuously introduced and approved. Recent efforts by the medical community to better serve the field have led to the approval of new emojis such as the anatomical heart and anatomical lung, and a more comprehensive set of emojis could be highly beneficial [47]. The current lack of medical emojis presents an important window of opportunity for clinicians and researchers to work toward a consensus and shape an optimized future for this communication modality. For example, dermatologists could introduce specific emojis to illustrate sun safety and sunscreen use for skin cancer prevention. Emojis to encourage melanoma awareness could be used in conjunction with current skin tone emojis to share information about skin health, thereby helping to address questions about sunscreen being unnecessary for darker skin. Creative applications, including displaying smiley or frowny social approval emoticons [35] on public sunscreen dispensers, could also be potentially effective in increasing dispenser use and positive reinforcement.

### Conclusions

The brisk evolution of modern technologies is continuously shaping our lives and health behaviors. The incorporation of emojis into communication has improved pediatric medicine, adult mood and psychological assessments, medication adherence, and public health tracking and interventions before and during the COVID-19 pandemic. Integration into dermatology practice has so far been limited but is ripe for expansion. Examining the surge in electronic communication that reaches new heights during the pandemic will be crucial to the continued advancement of health care. We aim to spark further innovation by highlighting the recent use and emerging ideas for emoji applications, and it will be intriguing to investigate future developments.

## Abbreviations

CPR: cardiopulmonary resuscitation
DSM-IV: Diagnostic and Statistical Manual of Mental Disorders–Fourth Edition
PHQ: Patient Health Questionnaire
